# High-performance thin-layer chromatography in combination with an acetylcholinesterase-inhibition bioassay with pre-oxidation of organothiophosphates to determine neurotoxic effects in storm, waste, and surface water

**DOI:** 10.1007/s00216-022-04068-6

**Published:** 2022-05-18

**Authors:** Nicolai Baetz, Torsten C. Schmidt, Jochen Tuerk

**Affiliations:** 1 Institut für Energie- und Umwelttechnik e. V. (IUTA, Institute of Energy and Environmental Technology), Bliersheimer Str. 58–60, 47229 Duisburg, Germany; 2 Instrumental Analytical Chemistry, Faculty of Chemistry, University of Duisburg-Essen, Universitätsstr. 5, 45141 Essen, Germany; 3 Center for Water and Environmental Research (ZWU), University of Duisburg-Essen, Universitätsstr. 2, 45141 Essen, Germany

**Keywords:** High-performance thin-layer chromatography (HPTLC), Acetylcholinesterase-inhibition assay (AChE-I assay), Oxidation by N-bromosuccinimide, Organothiophosphates

## Abstract

Pesticides such as organothiophosphates (OTPs) are neurotoxically active and enter the aquatic environment. Bioassays, using acetylcholinesterase (AChE), a suitable substrate and reactant, can be applied for the photometric detection of AChE-inhibiton (AChE-I) effects. The oxidized forms of OTPs, so-called oxons, have higher inhibition potentials for AChE. Therefore, a higher sensitivity is achieved for application of oxidized samples to the AChE assay. In this study, the oxidation of malathion, parathion, and chlorpyrifos by n-bromosuccinimide (NBS) was investigated in an approach combining high-performance thin-layer chromatography (HPTLC) with an AChE-I assay. Two AChE application approaches, immersion and spraying, were compared regarding sensitivity, precision, and general feasibility of the OTP effect detection. The oxidation by NBS led to an activation of the OTPs and a strong increase in sensitivity similar to the oxons tested. The sensitivity and precision of the two application techniques were similar, although the spray method was slightly more sensitive to the oxidized OTPs. The 10% inhibition concentrations (IC_10_) for the spray approach were 0.26, 0.75, and 0.35 ng/spot for activated malathion, parathion, and chlorpyrifos, respectively. AChE-I effect recoveries in samples from a stormwater retention basin and receiving stream were between 69 and 92% for malathion, parathion, and chlorpyrifos. The overall workflow, including sample enrichment by solid-phase extraction, HPTLC, oxidation of OTPs, and AChE-I assay, was demonstrated to be suitable for the detection of AChE-I effects in native water samples. An effect of unknown origin was found in a sample from a stormwater retention basin.

**Supplementary Information:**

The online version contains supplementary material available at 10.1007/s00216-022-04068-6.

## Introduction

The worldwide use of pesticides has caused a contamination of the environment in all compartments such as water bodies, soil, and air. Pesticides and biocides can harm and alter organisms, populations, and entire food webs in several ways. Some compounds are suspected of being carcinogenic or influencing hormone balance, so that residues in food and ground and drinking water can also pose a risk for humans [1]. Pesticides and biocides are introduced into the aquatic environment via runoff from agricultural areas, discharges from combined or separate sewer systems and wastewater treatment plants [2–5]. Biocides used in non-agricultural sectors can enter the environment, for example, when they evaporate or leach from facade painting [6, 7]. In addition, pesticide residues are found in food [8].

Organophosphates and carbamates are two widespread pesticide groups and have been analyzed in different matrices all the way from plants and soils to the aquatic system [9, 10]. These two groups of pesticides inhibit acetylcholinesterase (AChE), which occurs in the nervous system of mammals, birds, fish, reptiles, and insects [11–13]. Enzyme-based biosensors were used for neurotoxicity testing of pesticides in the environment [14]. The AChE activity can be measured by well-established AChE assays [15–18]. The combination of high-performance thin-layer chromatography (HPTLC) with AChE-I assays has been successfully demonstrated and used for effect-directed analysis (EDA) of environmental, food, and plant samples [19–28]. Organothiophosphates (OTPs), such as chlorpyrifos and malathion, become stronger AChE inhibitors when the sulfur atom in the phosphorus-sulfur bond is replaced biologically (metabolic or microbiological actions) or chemically (chemical or photo-oxidation) by an oxygen atom [11–13, 18, 19, 29]. The OTPs are not always completely oxidized to the respective oxons, but other products or a further transformation may take place [30, 31]. Higher inhibition sensitivities can be achieved by a pre-oxidation of OTPs whereby bromine is used by several authors before applying an AChE-I assay on HPTLC plates [19, 32, 33]. Other studies presented a biological activation of OTPs [34–36]. OTPs in lower inhibition concentrations are detectable and most important the metabolic OTP activation in organisms can be simulated in this way. N-Bromosuccinimide (NBS) is an alternative bromine containing oxidizing agent that is used in AChE-I assays and has proven to be suitable for a complete oxidation of OTPs in water samples [37, 38].

Two methods, immersion and spraying, for the application of biosensors on HPTLC plates have been used in a study by Azadniya and Morlock [39]. Mendoza et al. [20] and Stütz et al. [23] used, as in this study, indoxyl acetate as an esterase substrate but each of them employed a different method to apply the esterase solution on thin-layer plates: immersion or spraying. In a previous work, an immersion and a spray method for application of yeast suspension on HPTLC plates were compared whereas a similar sensitivity and a better precision of the spraying approach were observed [40]. Schoenborn et al. [41] sprayed yeast cells onto HPTLC plates and observed an unprecedented sensitivity of the planar yeast estrogen screen compared to immersion.

This study aims to demonstrate that oxidation of OTPs by NBS is successful not only in an AChE-I microtiter assay but also in a combination of HPTLC, oxidation, and AChE-I assay (HPTLC-Ox-AChE-I). An expected increase in sensitivity will be demonstrated by investigating dose–response relations of malathion, parathion, chlorpyrifos, and related oxons either with or without oxidation by NBS after chromatographic separation. Moreover, it is interesting to ask whether differences in sensitivity, precision, and general feasibility can be observed in an HPTLC-Ox-AChE-I approach when the enzyme is sprayed onto the HPTLC plates or the plates were immersed into the enzyme solution. As a proof of concept, native water samples from a stream and connected stormwater retention basin were investigated with the HPTLC-Ox-AChE-I method after enrichment by solid-phase extraction (SPE).

## Material and methods

### Chemicals

The pesticides parathion, chlorpyrifos, and malathion as well as the oxons paraoxon and malaoxon were purchased from Sigma-Aldrich GmbH (Steinheim, Germany). Chlorpyrifos-oxon was purchased from LGC standards GmbH (Wesel, Germany). All standards had a purity of > 95%. AChE from electric eel (*Electrophorus electricus*), acetylthiocholine (ATCL), NBS, 5,5′-dithiobis(2-nitrobenzoic acid) (DTNB), ascorbic acid, and bovine serum albumin (BSA) were purchased from Sigma-Aldrich GmbH (Steinheim, Germany). Tris(hydroxymethyl)aminomethane (TRIS) was purchased from Carl Roth GmbH + Co. KG (Karlsruhe, Germany). HCl was purchased from Merck (Darmstadt, Germany). Indoxyl acetate was purchased from Thermo Fisher Scientific (Geel, Belgium). Methanol (LC–MS grade), acetone (LC–MS grade), dichloromethane (LC–MS grade), and water (LC–MS grade) were all purchased from Th. Geyer GmbH & Co. KG (Renningen, Germany). Cyclohexane (LC–MS grade) was purchased from LGC Standards GmbH (Wesel, Germany).

### AChE-inhibition microtiter assay

Stock solutions (1 mg/mL) of NBS and ascorbic acid were each freshly prepared before the test and then diluted to 10 µg/mL and 100 µg/mL, respectively. The assay was performed with modifications according to Ellman’s method [15]. OTPs, oxons, water, and methanol blanks (15 µL) were each mixed with 15 µL of NBS (10 µg/mL) in a 96-well plate. After an incubation at room temperature for 5 min, the reaction was stopped by adding 15 µL ascorbic acid (100 µg/mL). For the test setup without oxidation, OTPs, oxons, water, and methanol blanks (15 µL) were mixed with 30 µL water per well. Subsequently, 200 µL of a DTNB solution (0.15 mM), buffered in TRIS/HCl at a pH of 7.4, was added to each well of both test setups. Then, 20 µL of an AChE solution (0.5 U/mL), also buffered in TRIS/HCl at a pH of 7.4, was added. An incubation at 30 °C for 30 min without shaking followed. An ATCL solution (0.8 mM) was freshly prepared with water and 30 µL per well was added after incubation. During another incubation at room temperature for 30 min, ATCL was cleaved by AChE into thiocholine and acetic acid. Thiocholine reacts with DTNB to 2-nitro-5-thiobenzoate [15]. The absorbance of the yellow-colored anion was photometrically measured at 405 nm (Sunrise Remote, Tecan Group AG, Männedorf, Schweiz). A flow chart of the used procedure is shown in Fig. S1.

#### Dose–response relationship

Dilution series with eight concentrations of the three OTPs and related oxons (Tab. S1) were freshly prepared from stock solutions (1–10 mg/mL) in methanol. The OTPs and oxons were tested in duplicates with and without prior oxidation in two measurements as described in the “AChE-inhibition microtiter assay” section. Eight water and eight methanol blanks were tested per test setup (oxidation and no oxidation). Of these, two were tested without AChE and two without ATCL. The mean AChE activity (absorption at 405 nm, *n* = 4) was related to the specific inhibitor dose. The statistic program Prism (version 5.00, Graph Pad Software, San Diego, USA) was used to generate dose–response curves (4-PL fit) and to determine inhibition concentrations (ICs) for the three OTPs and related oxons. The 95% confidence intervals of the curves and the standard deviations of the mean AChE activity were also calculated. The lower boundary of the curves is fixed to 0 and the highest mean activity defines 100%.

### Combination of HPTLC and AChE-inhibition assay

#### HPTLC

LiChrospher 10 × 20 cm HPTLC Silica gel 60 F254s plates (Merck, Darmstadt, Germany) with a layer thickness of 170–190 µm and spherical silica particles with a size of 7 µm were used. The HPTLC plates were immersed twice in 2-propanol for 20 min, each time followed by a drying step at room temperature for 20 min. The plates then were pre-developed with methanol, dried for 20 min at room temperature, heated at 105 °C for 20 min, and stored in a desiccator for further use. The automatic TLC Sampler 4 (ATS 4, CAMAG AG, Muttenz, Switzerland) was used for sample application. The desired sample volumes were sprayed as 6-mm-wide bands onto the HPTLC plates with an application speed of 300 nL/s. The distances of the exterior bands from the edges of the plates were 15 mm. The distance from the bottom edge was 8 mm for all bands. Methanol was used as rinsing solvent. The following further application parameters were used: filling speed 15 µL/s, pre-dosage volume 200 nL, retraction volume 200 nL, rinsing vacuum time 4 s, filling vacuum time 0 s, rinsing cycles 2, and filling cycles 1. The development of the HPTLC plates was realized by the Automated Multiple Development 2 (AMD 2, CAMAG, Muttenz, Switzerland). The OTPs, their oxons, and environmental samples were separated with an eluent mixture of cyclohexane, dichloromethane, and acetone in different proportions and with increasing migration distances (Fig. S2). After each development step, a drying step of 3 min under vacuum followed. The final migration distance was 80 mm and the whole procedure took approximately 45 min. In addition, a simpler development process was used containing only the last step of the previous described method.

#### Chemical oxidation of OTPs and AChE-inhibition assay

After chromatographic development, 10 mL of freshly prepared NBS (100 µg/mL) was sprayed onto the HPTLC plates with a glass reagent sprayer, followed by a 5-min incubation at room temperature. The following AChE-I assay was performed in parts according to Mendoza et al., Stütz et al., and Weins and Jork [20, 22, 42]. AChE was buffered in TRIS/HCl at a pH of 7.8 (2.5 U/mL). The AChE solution was either sprayed onto the HPTLC plates with a glass reagent sprayer until the plates were evenly moist (approx. 10 mL) or the plates were immersed in the AChE solution using the Chromatogram Immersion Device 3 (CID 3, CAMAG, Muttenz, Switzerland). The immersion volume, speed, time, and depth were approx. 200 mL, 2.5 cm/s, 2 s, and approx. 85 mm, respectively. Afterward, the plates were placed separately in closed plastic boxes, which contained paper towels moistened with water to gain a saturated atmosphere. An incubation at 37 °C for 5 min followed. The substrate indoxyl acetate was freshly prepared in methanol at a concentration of 20 mg/mL and 5 mL was sprayed onto the HPTLC plates with a glass reagent sprayer. During 45-min incubation time at room temperature, AChE cleaves the substrate into indoxyl and acetate. The indoxyl reacts with oxygen to the blue indigo dye. When AChE was inhibited, no indigo was produced and the spot stayed white. The TLC Scanner 3 (CAMAG, Muttenz, Switzerland) was used to scan each track on the plates at 670 nm using the fluorescence mode without optical filter. The CAMAG-embedded software Wincats (Vers. 1.4.9) was used to provide chromatograms of each track and to evaluate inhibition zones.

#### Separation of organothiophosphates and oxons by HPTLC

The mean retardation factors (*R*_F_s) and standard deviations (SDs) (*n* = 18) of the OTPs and oxons were calculated using Excel 2013 (vers. 15.0.5172.1000, Microsoft, Redmond, USA). For the four-step development process, the *R*_F_s and SDs from the different tested amounts of the dose–response investigations (see the “Dose–response relationship” section) were used (*n* = 18). The separation of OTPs and oxons with the single-step development was done in duplicate on two HPTLC plates (*n* = 4). The influence of the single- and four-step development on the migration of sample matrix was investigated by using a SPE extract from a combined sewer overflow.

#### Dose–response relationship

Several amounts of an OTP mix and an oxon mix were applied on HPTLC plates (Tab. S2) and tested as described in the “Combination of HPTLC and AChE-inhibition assay” section. Three plates were used for each dilution series. The application volume was 10 µL for investigating OTPs with following oxidation and 100 µL for testing OTPs without following oxidation. The application volume for the oxons was 10 µL. They were tested without oxidation step also in triplicates. The peak heights were used for evaluation. Only peaks that have a signal-to-noise ratio ≥ 3 were considered in the evaluation. The height next to the respective peak defines the noise. The first detected peak at one of the applied concentrations with a signal to noise ratio ≥ 3 defines the LOD for the specific substance and the respective application method. The mean AChE-I (mean peak height, AU at 670 nm, *n* = 3) was related to the inhibitor amounts. Prism was used to generate dose–response curves (4-PL fit) and to determine ICs for the three OTPs (oxidized and unoxidized) and related oxons. The 95% confidence intervals of the curves and the standard deviations of the mean peak heights were also calculated. The lower boundary of the curves is fixed to 0 and the highest mean peak height defines 100%.

### Proof of concept using environmental samples

#### Sampling

Grab samples were taken in March and August 2020 from a stormwater retention basin, which is connected to a highway nearby, and up- and downstream the receiving stream Deininghauser Bach near Deininghausen, Germany. A field blank was prepared with LC–MS grade water, which was used to wash the sampling vessel and opened at the sampling points. The samples were cooled during transport and stored < 8 °C until further sample preparation.

#### Sample preparation

The samples were enriched by SPE within 24 h after sampling. Moreover, the field blank and water for a SPE blank were enriched in the same way. For additional matrix investigations, 1 mL malathion, parathion, and chlorpyrifos (100 ng/mL in methanol) were spiked into native samples taken in August 2020 and a water control before being loaded on the SPE cartridges. The cartridges (150 mg, Oasis HLB 6 cc, Waters GmbH, Eschborn, Germany) were conditioned with methanol (2 × 5 mL) and equilibrated with water (2 × 5 mL), before being loaded with 1000 ± 5 mL sample (the exact volume was determined by weighting) through a polytetrafluoroethylene tube. After drying the cartridges under vacuum, they were stored at − 18 °C until further usage. The cartridges were eluted with methanol (5 × 5 mL) that was evaporated afterward at 60 °C under a gentle nitrogen gas stream. The dried extracts were redissolved in 1 mL methanol to achieve a nominal 1000-fold enrichment.

#### HPTLC and AChE-inhibition assay

The extracts, spiked samples, positive controls (PC: mix of malathion, parathion, and chlorpyrifos), and blanks were tested with the HPTLC-Ox-AChE-I method as described in the “Combination of HPTLC and AChE-inhibition assay” section. Each sample was tested in duplicates on two HPTLC plates (*n* = 4). The application volume was 100 µL resulting in a PC of 2000 ng/spot. The resulting amounts for the matrix investigation were 1000 and 10 ng/spot for the PCs and 10 ng/spot for the spiked samples. The matrix investigation was performed with and without the oxidation step. Excel was used to relate the peak areas of the spiked samples to the peak areas of the PCs (10 ng/spot). Differences between both approaches (oxidation and no oxidation) were shown by comparing the results of the spiked samples and PCs.

## Results and discussion

### AChE-inhibition microtiter assay

**Fig. 1:**
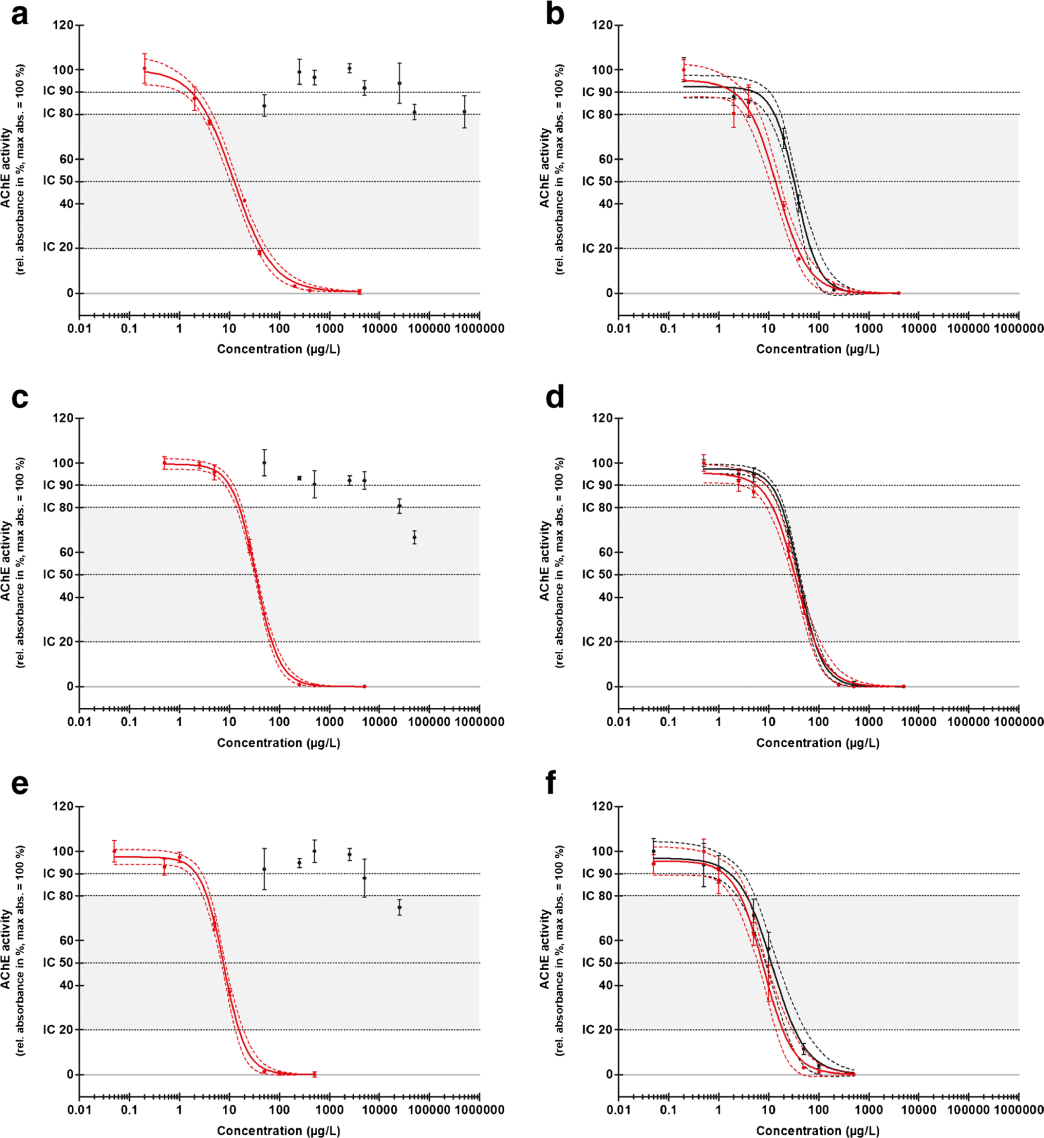
Dose–response curves (4-PL fit) of malathion (a, b), parathion (c, d), and chlorpyrifos (e, f) (black dots and curves), and malaoxon (a, b), paraoxon (c, d), and chlorpyrifos-oxon (e, f) (red dots and curves). An AChE inhibition assay for the detection of inhibition effects was performed in 96-well plates (AChE-I microtiter assay) either without (left side: a, c, and e) or after oxidation by N-bromosuccinimide (right side: b, d, and f). Acetylthiocholine was used as substrate and DTNB as reactant for thiocholine. The AChE activity (relative absorbance in %) is shown on the *y*-axis and the specific substance concentration (µg/L) on the *x*-axis (*n* = 4). The lower boundary of the curves is fixed to 0. The highest mean absorbance defines 100%. The dashed lines show the 95% confidence intervals, and the error bars the standard deviations. The dotted lines indicate the inhibition concentration (IC) for 90, 80, 50, and 20% AChE activity. The grey-colored area between IC_80_ and IC_20_ shows the linear range

In a preliminary experiment using microtiter plates, it was confirmed that oxidation with NBS is suitable to increase the AChE-I potential of the tested OTPs, and in this way, the sensitivity of AChE assays to OTPs as shown in other studies [36, 43]. Although concentrations up to 500 µg/mL were used, the inhibition intensities of the unoxidized OTPs were not high enough to generate full sigmoidal dose–response curves in contrast to oxidized OTPs (Fig. 1).

The observed differences of the ICs between oxons and oxidized OTPs could be due to an incomplete oxidation of OTPs to their corresponding oxons, a formation of other non-active products, or a decay of formed oxons. Kralj et al. [37] showed that with an increasing NBS concentration formed oxons could not be detected or the signals decreased, which could be an indication for a further transformation of the oxons. In a further study by Kralj et al. [31], it is shown that several transformation products were formed during photolysis or photocatalysis of malathion.

The increase in toxicity of OTPs due to biotransformation during natural metabolism can be mimicked in a simple way by using NBS for chemical oxidation. Biological activation of OTPs with cytochrome P450 proteins is maybe closer to the metabolism in vivo. However, in vivo metabolism depends on different cytochrome P450 types and differs between organisms and could be even sex-specific [44, 45]. Studies often used one enzyme for activation of OTPs such as a genetically engineered P450 or chloroperoxidase [35, 36, 46]. In comparison, the chemical oxidation process presented is much less demanding in terms of preparation, chemicals, and materials. In addition, biological activation requires a buffer with a pH value that is within the optimum of the enzyme used for oxidation which does not necessarily match a subsequent AChE-I microtiter assay. Thus, the used oxidation method is a simpler and more robust procedure for testing native water samples. The sensitivity of the following HPTLC-Ox-AChE-I approach was in a comparable range to the AChE-I microtiter assay. However, the application volume of the HPTLC-Ox-AChE-I approach is decisive, so that better sensitivities can also be achieved. The IC_50_ of malathion, parathion, and chlorpyrifos for the microtiter assay were 35, 41, and 12 µg/L, respectively (Tab. S3). In comparison, when 100-µL sample was applied onto the HPTLC plate, the IC_50_ of malathion, parathion, and chlorpyrifos were 14, 42, and 15 µg/L, respectively.

### High-performance thin-layer chromatography

For the dose–response investigations and following analysis of environmental samples, a development method was needed that is able to separate malathion, parathion, chlorpyrifos, and the related oxons on the HPTLC plate. With the four-step development, it was possible to separate all three OTPs and the oxons to a sufficient extent for the following investigations (Table 1 and Fig. S4). The single-step method allowed a separation of all 6 compounds with reduced time, cost, and solvent consumption (Tab. S5). In addition, attempts were made to better separate polar matrix of environmental samples from non-polar fractions. For this purpose, an unconventional approach, the four-step HPTLC development procedure with increasing migration distance and increasing elution power (Fig. S2), was compared to the single-step development method. A sample extract from a combined sewer overflow (CSO) was separated by the four-step and the single-step development procedures. The four-step development showed less migration of visible sample components in comparison to the single-step method before applying the AChE-I assay (Fig. S5). Using the four-step development, the sample matrix had less influence on at least the last third of the solvent migration distance in contrast to the single-step development. Whether matrix retention on a HPTLC plate can actually be achieved with such a development and what benefit it has over single-step or multi-step development with increasing migration distance and decreasing elution power should be determined in further, more in-depth studies. However, the four-step development was used for the following dose–response experiments and analysis of water samples because a sufficient separation of the OTPs was achieved and an influence of the separation method on the results of dose–response investigations is rather unlikely.

**Table 1:** The mean retardation factor (*R*_F_), standard deviation (SD), calculated relative *R*_F_, and resolution *R* of malaoxon, paraoxon, chlorpyrifos-oxon, malathion, parathion, and chlorpyrifos on the LiChrospher HPTLC plate. After chromatographic separation, using a four-step HPTLC development process, and oxidation by N-bromosuccinimide, the plates were measured with an AChE inhibition assay using indoxyl acetate as substrate. The HPTLC plates were either immersed in AChE solution (left side) or AChE was sprayed onto the plates (right side). The plates were scanned at 670 nm. The mean *R*_F_s and SDs were calculated from the different tested amounts (1–250 ng/spot) of the dose–response investigations (*n* = 18)

Substances	Immersion method				Spray method			
	Mean *R*_F_	SD	rel. *R*_F_	*R*	Mean *R*_F_	SD	rel. *R*_F_	*R*
Malaoxon	0.22	0.04			0.21	0.02		
Paraoxon	0.31	0.04	1.4	1.0	0.29	0.02	1.4	1.1
Chlorpyrifos-oxon	0.50	0.07	1.6	1.9	0.47	0.03	1.6	2.2
Malathion	0.74	0.06	1.5	2.2	0.67	0.03	1.4	2.1
Parathion	0.85	0.03	1.2	1.3	0.78	0.04	1.2	1.3
Chlorpyrifos	0.93	0.02	1.1	1.0	0.85	0.05	1.1	1.0

### Oxidation on high-performance thin-layer plates

**Fig. 2:**
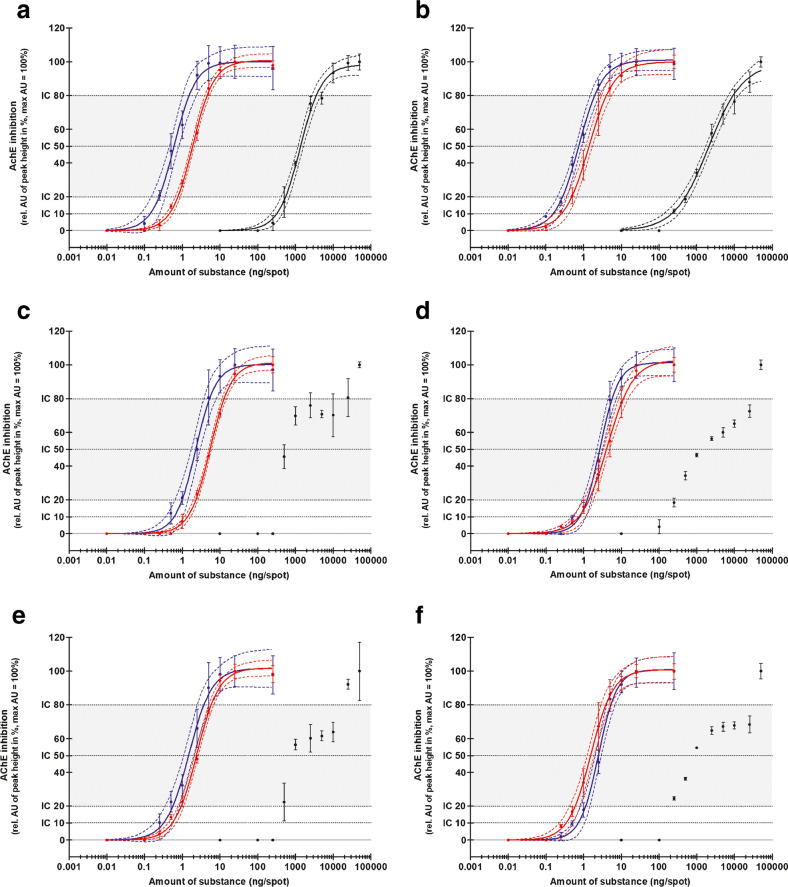
Dose–response curves (4-PL fit) of different organothiophosphates (OTPs) and their oxons. An OTP mix and an oxon mix were applied with different concentrations on LiChrosphere HPTLC plates. The application volume was 10 μL for the oxons and OTPs with following oxidation and 100 µL for the OTPs without following oxidation. After chromatographic separation by HPTLC, an AChE inhibition assay either with or without prior oxidation by N-bromosuccinimide was performed (HPTLC-(Ox)-AChE-I). The HPTLC plates were either immersed in AChE solution (a, c, e) or AChE was sprayed onto the plates (b, d, f). The black, red, and green dots and curves represent the AChE inhibition by malathion, oxidized malathion, and malaoxon (a, b); parathion, oxidized parathion, and paraoxon (c, d); and chlorpyrifos, oxidized chlorpyrifos, and chlorpyrifos-oxon (e, f), respectively. The peak heights were used for evaluation. Only peaks that have a signal-to-noise ratio ≥ 3 were considered in the evaluation. The height next to the respective peak defines the noise. The AChE inhibition is shown on the *y*-axis as the relative AU of the mean peak heights (*n* = 3) at a specific amount of substance on the HPTLC plate (*x*-axis). The lower boundary of the curves is fixed to 0. The highest mean peak height defines 100%. The dashed lines show the 95% confidence intervals and the error bars the standard deviations. The dotted lines indicate the inhibition concentration (IC) for 10, 20, 50, and 80% AChE inhibition. The grey-colored area between IC_20_ and IC_80_ shows the linear range

The dose–response relations of malathion, parathion, chlorpyrifos, and the related oxons were used to evaluate the oxidation by NBS on the HPTLC plate. In Fig. 2, Tab. S4, and Tab. S6, it is obvious that the oxidation of the three OTPs on HPTLC plates can be applied as expected. The sensitivity of the HPTLC-AChE-I assay can be increased dramatically for the detection of OTPs by oxidation with NBS. Differences between the AChE-I potential and thus the ICs of the tested oxons and the activated OTPs (Fig. 2, Fig. S4, and Tab. S4) could be due to an insufficient oxidation of the OTPs on the HPTLC plate. Another possibility is, as discussed for the AChE-I microtiter assay, a formation of other OTP products or a partial decay of the formed oxons during the following incubation times. The final scan of the HPTLC plate takes place about an hour after the application of NBS. Akkad et al. [33] used bromine for oxidation before applying a HPTLC esterase inhibition assay (HPTLC-EI) and showed an increase of the inhibition potential of OTPs. They also observed differences of the esterase inhibition between the activated OTPs and corresponding oxons. Azadniya et al. [34] used an S9 mixture to metabolize OTPs successfully on HPTLC plates. After sample application, a pre-wetting step follows before they applied the S9 mixture. An incubation of 30 min followed. The benefit of the here presented oxidation method with NBS is its simplicity: one application step followed by an incubation of 5 min. For a further increase of the sensitivity, a higher amount of NBS for oxidation could be reasonable. However, a concentration of 1 mg/mL NBS was tested, resulting in a lower activity or inhibition of AChE. Less indoxyl acetate seemed to be cleaved by AChE, and therefore, less indigo was formed. The HPTLC plate only became a blue tinge instead of a stronger blue color.

### Comparison between immersion and spray methods

The comparison between immersion and spray approaches revealed that when using the immersion method to apply AChE on the HPTLC plates, the OTPs showed significantly higher *R*_F_s than using the spray approach (D’Agostino and Pearson omnibus normality test and Mann–Whitney test) (Table 1). A reason could be that the immersion method influences the migration retrospectively. However, this assumption is counteracted by the fact that the *R*_F_s of the oxons do not differ significantly from each other. Notable differences in the effect peaks’ width were not observed between immersion and spray methods, which would have to be assumed if the immersion method had an influence. Since the immersion line (85 mm) is quite close to the solvent front (80 mm) and the OTPs again have very high *R*_F_ values, a shift of the OTPs may have occurred in the upper part of the plate near the immersion line. After using a combination of HPTLC and enzyme inhibition assay in which plates were immersed in the enzyme solution, Akkad and Schwack [21] showed that some OTP spots with very high *R*_F_ values were more blurred than compounds with *R*_F_ values less than 0.5.

The comparison between immersion and spray approaches with the dose–response investigations showed that the spray method had a slightly better sensitivity to all tested activated OTPs (Fig. 2, Tab. S4, and Tab. S6). Low concentrations are thus more likely to be detected. The IC_10_s substantiate that. In another study, the application of yeast cells on the HPTLC plate for the detection of endocrine effects was compared [40]. An overall similar sensitivity was shown between spray and immersion approaches. Schoenborn et al. [41] observed a better sensitivity of the planar yeast estrogen screen by spraying yeast suspension onto HPTLC plates. One reason for a better sensitivity of spray methods is maybe that substances such as OTPs could be extracted to a small extent from the HPTLC plates during the immersion process. Schoenborn et al. [41] obtained higher peak quality with the spray method and blurred peaks with the immersion approach. A difference in the peak quality such as wider peaks with the immersion than with the spray method could not be observed in this study. The linear ranges between IC_20_ and IC_80_ are suitable for calibration. A comparison of the precision between the two application methods does not give a consistent picture. The 95% confidence intervals of the dose–response curves were narrower for the activated OTPs when using the immersion method, but wider for the oxons, in comparison to the spray method (Fig. 2, Tab. S4). In a previous study, investigating the application of a yeast suspension, the spray approach was slightly more precise than the immersion method [40]. Differences were the use of an airbrush to apply the yeast suspension onto the HPTLC plate until it was evenly moist. In this study, a glass reagent sprayer was used to spray the AChE solution onto the HPTLC plate until it was evenly wet, almost as with the immersion method, to guarantee an even distribution. Azadniya and Morlock compared immersion and piezoelectric spraying for application of cholinesterase and substrate solutions on HPTLC plates [39]. Besides many advantages of the spray method, they reported that the main argument in favor of the immersion method is its simplicity. This was also the case in this study because complete wetting of the HPTLC plates took much longer than it did with the immersion method. The spraying method was therefore less user-friendly, which could be counteracted by automatic spraying methods [39, 47]. Despite the slight differences in sensitivity, precision, and handling, both application methods behave very similar and can be used for the detection of AChE-I effects in an HPTLC-Ox-AChE-I approach. The spray approach was used for the following proof of concept with environmental samples because of the slightly higher sensitivity and the associated higher probability of detecting effects in low concentration ranges.

### Environmental samples

Samples taken from a stream and a connected stormwater retention basin (see the “Sampling” section) were analyzed using the developed HPTLC-Ox-AChE-I method including an enrichment by SPE. Besides the investigation of unknown AChE inhibition effects, the samples taken in August 2020 were spiked with malathion, parathion, and chlorpyrifos to show the recovery of the effects triggered by the OTPs. No notable differences between the stream and stormwater basin matrix were observed (Table 2). Malathion showed the highest recovery of the effect peak area related to the tested positive control. The recoveries in comparison to the positive control were between 81 and 92% for Malathion after SPE and HPTLC-Ox-AChE-I. The recoveries of parathion and chlorpyrifos were between 75–82% and 69–78%, respectively. A study by Akkad and Schwack, who combined HPTLC with a multi-enzyme inhibition assay with prior oxidation of organophosphates, showed recoveries of 91–106% for parathion, chlorpyrifos, and paraoxon in apple juice and tap water samples [33]. No notable matrix effects of the native samples could be shown in comparison to an ultrapure water sample spiked with the OTPs (Table 2). A reason for partly lower recoveries in the ultrapure water sample could be the organic material in the native samples that was retained in the cartridge and to which additional molecules bound. The spiked samples were also tested without oxidation. Neither the spiked water samples nor the positive control (10 ng/spot) showed any effect peaks. Thus, the oxidation by NBS works with native surface water sample extracts on HPTLC plates. If the samples were oxidized, the PC concentration (1000 ng/spot) is in the maximum inhibition plateau (Fig. 2). At this level, the peak heights do not increase but the areas continue to increase. Therefore, the peak area was used for evaluation. Only the positive control with 1000 ng/spot showed peaks for all three OTPs, but with lower intensities than the same positive control tested with oxidation step. The peak areas differed by a factor of 5–8 (Fig. S3). This shows that the chemical oxidation by NBS on HPTLC plates is a robust way to mimic the natural activation of OTPs by biotransformation.

**Table 2:** Recovery of the effect peak area of malathion, parathion, and chlorpyrifos. Native water samples and a water control sample were spiked with 100 ng of each organothiophosphate (OTP). The samples were taken at the Deininghauser Bach near Deininghausen next to the highway A42 from a rainwater retention basin, and up- and downstream the outlet of the rainwater retention basin in August 2020. After enrichment by solid-phase extraction (SPE), 100 μL of the extracts and the positive control (mix of the OTPs) were applied on LiChrosphere HPTLC plates, resulting in an OTP amount of 10 ng/spot. After chromatographic separation by HPTLC, an AChE inhibition assay with prior oxidation by N-bromosuccinimide was performed. AChE was sprayed onto the HPTLC plates. The mean peak areas of the spiked samples were related to the mean peak areas of the positive controls (100%). The recoveries ± standard deviations (%) are shown (*n* = 4)

Sampling point	Malathion	Parathion	Chlorpyrifos
Upstream	92 ± 11	82 ± 10	78 ± 7
Stormwater basin	81 ± 8	75 ± 9	74 ± 5
Downstream	87 ± 12	79 ± 13	69 ± 5
Spiked water	85 ± 6	74 ± 3	63 ± 4

**Fig. 3:**
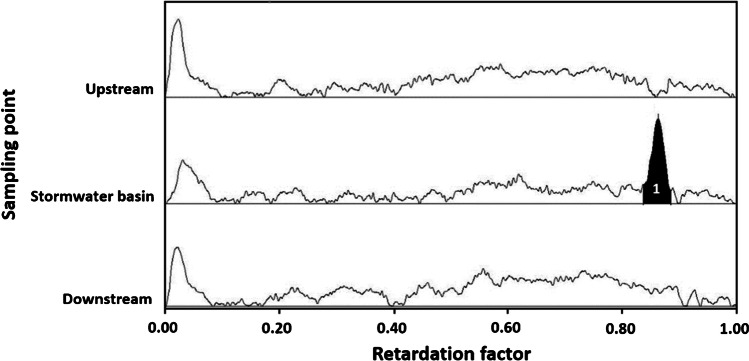
AChE inhibition effect (1) in the sample of a stormwater retention basin. The samples were taken at the Deininghauser Bach near Deininghausen, Germany next to the highway A42 from a stormwater retention basin, and up- and downstream the outlet of the stormwater basin in March 2020. After enrichment by solid-phase extraction (SPE), 100 μL of the extracts was applied on LiChrosphere HPTLC plates. As a positive control, a mix of malathion, parathion, and chlorpyrifos (each 2000 ng/spot) was also applied. After chromatographic separation by HPTLC, an AChE inhibition assay with prior oxidation by n-bromosuccinimide was performed. AChE was sprayed onto the HPTLC plates. The sampling points are shown on the *y*-axis and the retardation factor on the *x*-axis

The results showed that the overall workflow with prior sample enrichment by SPE is suitable to detect AChE-I effects caused by OTPs in surface water samples. This was confirmed by an AChE-I effect detected in an unspiked stormwater basin sample taken in March 2020 caused by unknown substances (Fig. 3). The samples taken in August 2020 showed no AChE-I effects. The presented HPTLC-Ox-AChE-I approach should facilitate subsequent analysis, because the complexity of the sample is reduced by HPTLC and information about the effect type are available, which reduces the number of possible responsible substances.

## Conclusion

This study demonstrates that an oxidation of OTPs by NBS can be applied in an HPTLC-Ox-AChE-I approach and leads to a strong increase of the substances’ inhibition potential and therefore a better sensitivity of the method. Chemical oxidation by NBS mimics the natural activation of OTPs in a more simple and robust manner than biological approaches, which allows a rapid investigation of neurotoxicity. The formation of different products than the corresponding oxons and the temporal processes and pathways during the oxidation should be investigated in detail for example by comparing dose–response relations of oxons that undergo the oxidation process or not. The comparison of the two main application methods for AChE on HPTLC plates, immersion and spraying, showed slight differences in sensitivity and precision. Both methods are suitable for the presented HPTLC-Ox-AChE-I approach. The potential of the spray approach for higher sensitivities should be refined. It may be reasonable to replace the manual spray approach by an automated device. The automation of the entire workflow may increase the reproducibility and comparability and in addition could save time and material. In conclusion, the combination of sample enrichment by SPE, separation by HPTLC, oxidation of possible OTPs, and AChE-I effect testing can be used to investigate neurotoxic activities in surface water samples. The overall workflow should be used to monitor these effects in the aquatic environment in more extensive studies and for the clarification of unknown effect responsible substances in combination with further instrumental analysis.

## Supplementary Information

Below is the link to the electronic supplementary material.

Supplementary file1 (PDF 901 KB)

## Data Availability

The datasets generated during and analyzed during the current study are available from the corresponding author on reasonable request.
