# Supplementary material for: High-performance thin-layer chromatography in combination with an acetylcholinesterase-inhibition bioassay with pre-oxidation of organothiophosphates to determine neurotoxic effects in storm, waste, and surface water
Source: Anal Bioanal Chem. 2022 May 18;414(14):4167–78. doi: 10.1007/s00216-022-04068-6 (PMC9124651; doi:10.1007/s00216-022-04068-6)
Supplement: Supplementary file 1 — Supplementary file1 (PDF 901 KB) [file 216_2022_4068_MOESM1_ESM.pdf]

Supplementary Information

High-Performance Thin-Layer Chromatography in Combination with an Acetylcholinesterase Inhibition Bioassay with Pre-Oxidation of Organothiophosphates to Determine Neurotoxic Effects in Storm-, Waste-, and Surface water

Nicolai Baetz[1,2,3], Torsten C. Schmidt[2,3], Jochen Tuerk[1, 3,*]

[1] Institut für Energie- und Umwelttechnik e. V. (IUTA, Institute of Energy and Environmental Technology), Bliersheimer Str. 58 – 60, 47229 Duisburg, Germany

[2] Instrumental Analytical Chemistry, Faculty of Chemistry, University of Duisburg-Essen, Universitätsstr. 5, 45141 Essen, Germany

[3] Center for Water and Environmental Research (ZWU), University of Duisburg-Essen, Universitätsstr. 2, 45141 Essen, Germany

* Corresponding Author

Phone: +49 (0) 2065 418 - 179, Fax: +49 (0) 2065 418 - 211, Email: tuerk@iuta.de, Address: Institut für Energie- und Umwelttechnik e. V., (IUTA, Institute of Energy and Environmental Technology) Bliersheimer Str. 58 - 60, 47229 Duisburg, Germany

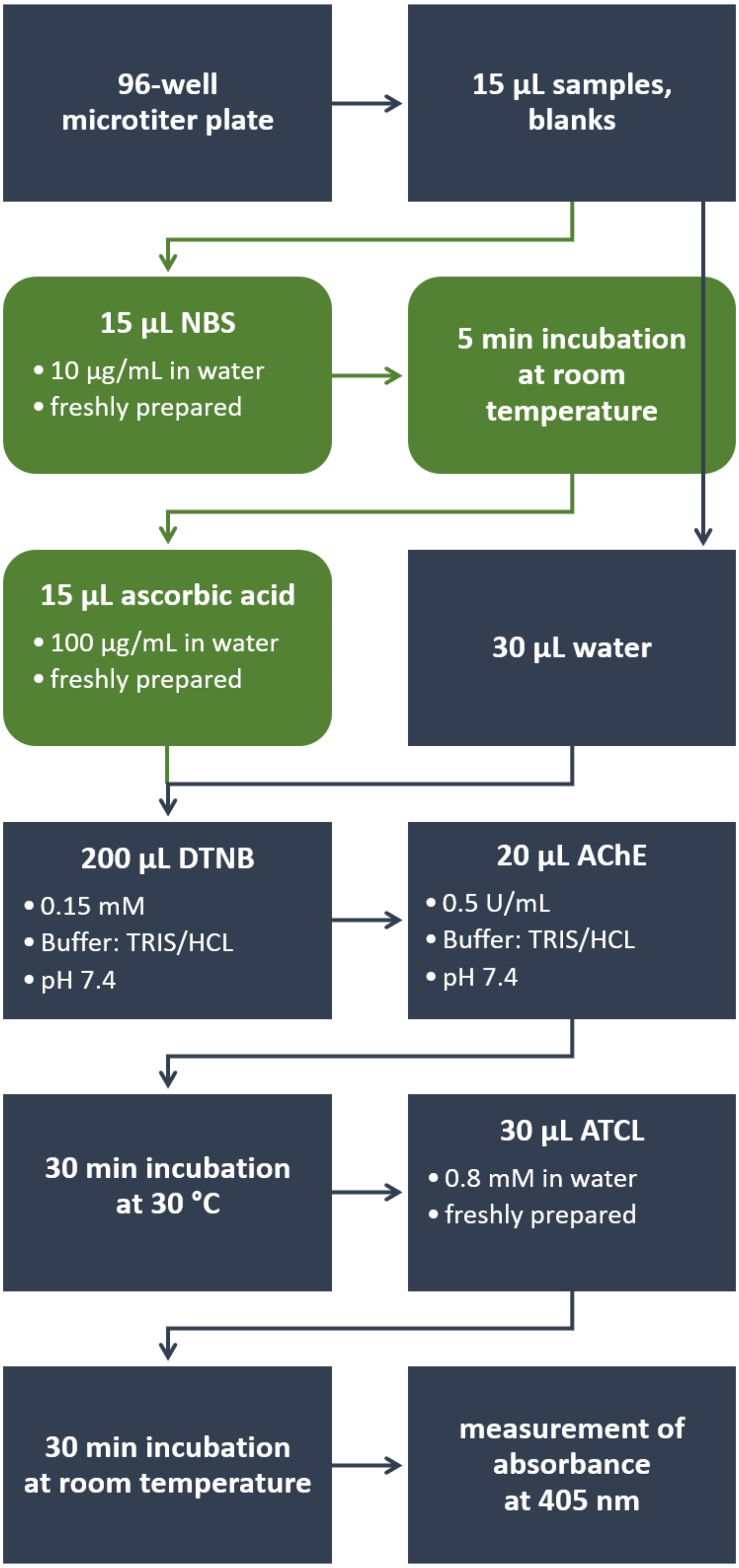

Fig. S1 Flow chart of the acetylcholinesterase inhibition (AChE-I) assay. The green steps show the additional oxidation with n-bromosuccinimide (NBS). Ascorbic acid stops the oxidation. Acetylthiocholine (ATCL) is cleaved by AChE into thiocholine and acetic acid. Thiocholine reacts with 5,5′-dithiobis (2-nitrobenzoic acid) (DTNB) to the yellow colored 2-nitro-5-thiobenzoate.

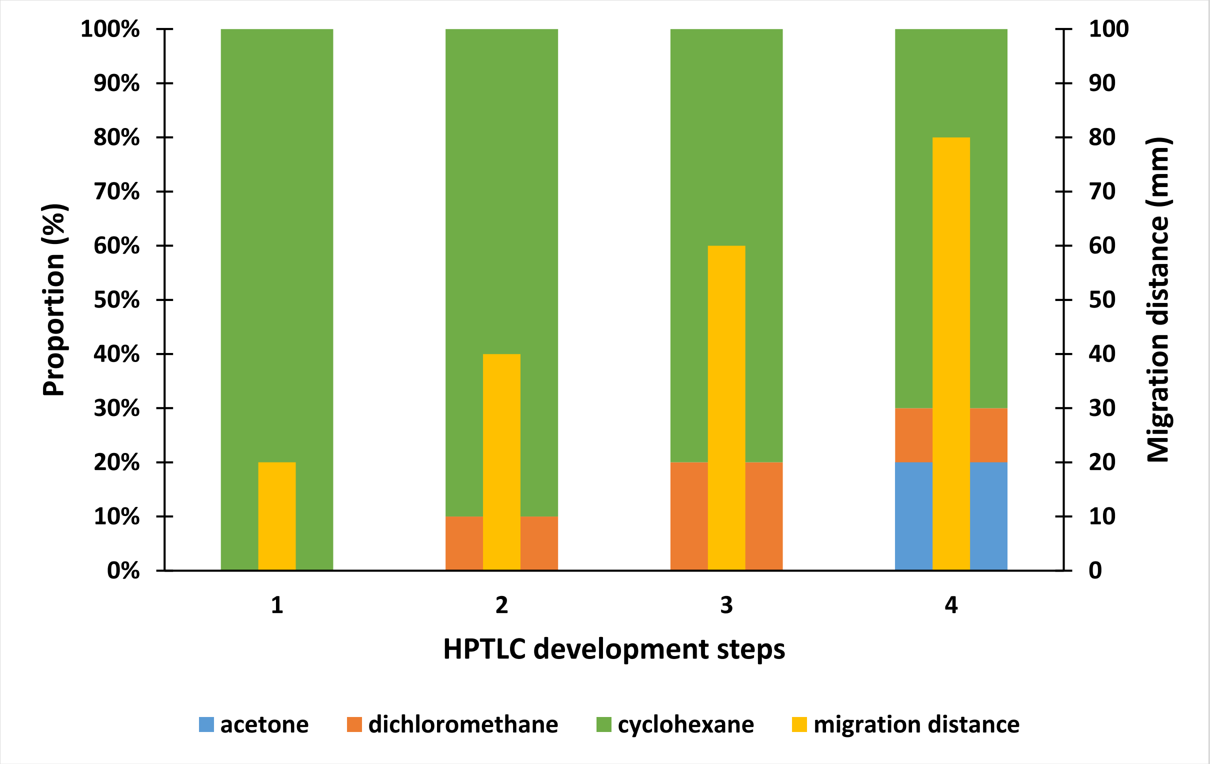

Fig. S2 Automated four-step HPTLC development process for separation on LiChrospher HPTLC plates. Every step contains another amount of the used solvents. First step: 100% cyclohexane, second step: 90% cyclohexane and 10% dichloromethane, third step: 80% cyclohexane and 20% dichloromethane, and fourth step: 70% cyclohexane, 10% dichloromethane, and 20% acetone. The green bars represent cyclohexane, the orange bars dichloromethane, and the blue bar acetone. The small yellow bars show the increasing migration distances. Final migration distance was 80 mm.

Tab. S1 Concentrations (µg/L) for parathion, chlorpyrifos, and malathion for dose-response investigations. The same concentrations as for the oxidized organothiophosphates were used for the specific oxons. An AChE assay for the detection of inhibition effects was performed in 96-well plates either without or with prior oxidation by N-bromosuccinimide. Acetylthiocholine was used as substrate and DTNB as reactant for thiocholine.

Parathion [µg/L]		Chlorpyrifos [µg/L]		Malathion [µg/L]	
**Non-oxidized**	**Oxidized**	**Non-oxidized**	**Oxidized**	**Non-oxidized**	**Oxidized**
50	0.5	50	0.05	50	0.2
250	2.5	250	0.5	250	2
500	5	500	1	500	4
2,500	25	2,500	5	2,500	20
5,000	50	5,000	10	5,000	40
25,000	250	25,000	50	25,000	200
50,000	500	50,000	100	50,000	400
500,000	5,000	500,000	500	500,000	4,000

Tab. S2 Concentrations (ng/spot) for parathion, chlorpyrifos, malathion, paraoxon, chlorpyrifos-oxon, and malaoxon for dose-response investigations. An organothiophosphate (OTP) mix and an oxon mix were applied with different concentrations on LiChrosphere HPTLC plates. The application volume was 10 µL for the oxons and OTPs with following oxidation and 100 µL for the OTPs without following oxidation. After chromatographic separation by HPTLC, an AchE inhibition assay either with or without prior oxidation by N-bromosuccinimide was performed. The HPTLC plates were either immersed in AchE solution or AchE was sprayed onto the plates.

**Organothiophosphates [ng/spot]**		**Oxons [ng/spot]**
**Non-oxidized**	**Oxidized**	
10	0.01	0.01
100	0.10	0.10
250	0.25	0.25
500	0.50	0.50
1,000	1.0	1.0
2,500	2.5	2.5
5,000	5.0	5.0
10,000	10	10
25,000	25	25
50,000	250	250

Tab. S3 Results of dose-response investigations for malathion, malaoxon, parathion, paraoxon, chlorpyrifos, and chlorpyrifos-oxon. An AChE assay for the detection of inhibition effects was performed in 96-well plates either without (left side) or with (right side) prior oxidation by N-bromosuccinimide. Acetylthiocholine was used as substrate and DTNB as reactant for thiocholine. The best-fit inhibition concentrations (IC) in µg/L for 90, 80, 50 and 20% AChE activity and the 95% confidence intervals of the dose-response curves (4-PL fit) are shown. The used concentrations of malathion, parathion, and chlorpyrifos were not sufficient for full dose-response curves without oxidation. No IC values could be calculated.

**Substance**		**No oxidation (c = µg/L)**				**Oxidation (c = µg/L)**			
		**IC_{90}**	**IC_{80}**	**IC_{50}**	**IC_{20}**	**IC_{90}**	**IC_{80}**	**IC_{50}**	**IC_{20}**
**Malathion**	Best fit	n/a	n/a	n/a	n/a	11	17	35	72
	95% confidence interval	n/a	n/a	n/a	n/a	7.0-17	12-23	30-41	52-100
**Malaoxon**	Best fit	1.7	3.5	12	42	3.0	5.4	15	39
	95% confidence interval	0.98-2-9	2.3-5-4	9.6-16	34-53	1.5-6.1	3.2-9.2	11-19	30-51
**Parathion**	Best fit	n/a	n/a	n/a	n/a	13	20	41	84
	95% confidence interval	n/a	n/a	n/a	n/a	11-16	17-23	38-44	74-96
**Paraoxon**	Best fit	10	16	34	71	8.4	14	35	86
	95% confidence interval	8.2-13	14-19	31-36	62-80	5.4-13	10-19	30-40	68-109
**Chlorpyrifos**	Best fit	n/a	n/a	n/a	n/a	2.2	4.1	12	34
	95% confidence interval	n/a	n/a	n/a	n/a	1.1-4.4	2.5-6.8	8.6-16	21-55
**Chlorpyrifos-oxon**	Best fit	2.5	3.8	7.7	16	1.8	3.1	7.9	20
	95% confidence interval	1.9-3.3	3.1-4.6	7.0-8.5	13-19	0.97-3.5	2.0-4.9	6.3-9.8	13-29

Tab. S4 Results of dose-response investigations for malathion, malaoxon, parathion, paraoxon, chlorpyrifos, and chlorpyrifos-oxon. An organothiophosphate (OTP) mix and an oxon mix were applied with different concentrations on LiChrosphere HPTLC plates. The application volume was 10 µL for the oxons and OTPs with following oxidation and 100 µL for the OTPs without following oxidation. After chromatographic separation by HPTLC, an AChE inhibition assay either with or without prior oxidation by N-bromosuccinimide was performed. The HPTLC plates were either immersed in AchE solution (left side) or AchE was sprayed onto the plates (right side). The peak heights were used for evaluation. Only peaks that have a signal-to-noise ratio ≥ 3 were considered in the evaluation. The heights next to the respective peak defines the noise. The best-fit inhibition concentrations (IC) in ng/spot for 10, 20, 50, and 80% AChE inhibition and the 95% confidence intervals of the dose-response curves (4-PL fit) are shown.

**Substance**		**Immersion method (ng/spot)**				**Spray method (ng/spot)**			
		**IC$_{10}$**	**IC$_{20}$**	**IC$_{50}$**	**IC$_{80}$**	**IC$_{10}$**	**IC$_{20}$**	**IC$_{50}$**	**IC$_{80}$**
**Malathion**	Best fit	313	536	1343	3363	212	493	2096	8910
	95% confidence interval	221–443	418–686	1,088–1,657	2,334–4,846	138–325	365–666	1,474–2,980	4,715–16,837
**Oxidized malathion**	Best fit	0.45	0.76	1.9	4.7	0.26	0.48	1.4	4.0
	95% confidence interval	0.36–0.56	0.66–0.89	1.7–2.1	3.8–5.8	0.17–0.40	0.35–0.66	1.1–1.8	2.5–6.4
**Malaoxon**	Best-fit	0.14	0.24	0.60	1.5	0.16	0.28	0.75	2.0
	95% confidence interval	0.08–0.26	0.16–0.37	0.44–0.83	0.85–2.7	0.10–0.24	0.21–0.37	0.60–0.94	1.4–3.1
**Oxidized chlorpyrifos**	Best fit	0.55	0.95	2.4	6.2	0.35	0.60	1.6	4.0
	95% confidence interval	0.43–0.69	0.80–1.1	2.1–2.8	4.9–7.9	0.22–0.53	0.44–0.82	1.2–2.0	2.5–6.2
**Chlorpyrifos-oxon**	Best fit	0.32	0.57	1.5	4.0	0.72	1.1	2.5	5.4
	95% confidence interval	0.17–0.59	0.36–0.88	1.0–2.2	2.1–7.4	0.49–1.1	0.86–1.5	2.0–3.1	3.8–7.7
**Oxidized parathion**	Best fit	1.4	2.3	5.6	13	0.75	1.4	4.2	13
	95% confidence interval	1.2–1.7	2.0–2.7	5.0–6.2	11–16	0.49–1.2	1.1–1.9	3.2–5.5	7.8–20
**Paraoxon**	Best fit	0.60	0.99	2.3	5.4	0.79	1.2	2.7	5.9
	95% confidence interval	0.34–1.1	0.66–1.5	1.7–3.2	3.1–9.2	0.53–1.2	0.94–1.7	2.2–3.3	4.2–8.4

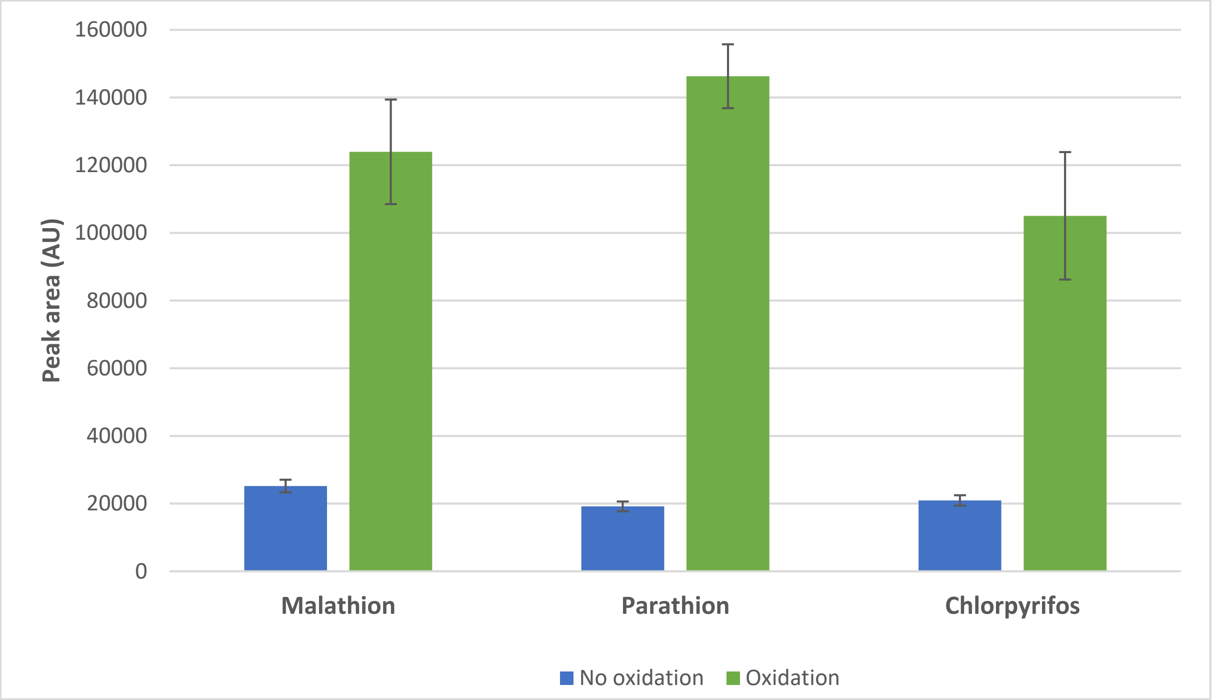

Fig. S3 Peak areas (AU) of positive controls containing malathion, parathion, and chlorpyrifos. After chromatographic separation by HPTLC, an AChE inhibition assay with (green bars) and without (blue bars) prior oxidation by N-bromosuccinimide was performed. AChE was sprayed onto the HPTLC plates. 10 µL of the positive control was applied in duplicates on two LiChrosphere HPTLC plates per method, resulting in 1000 ng/spot. The error bars represent the standard deviations.

Tab. S5 The mean retardation factor (R_F), standard deviation (SD), calculated relative R_F, and resolution R of malaoxon, paraoxon, chlorpyrifos-oxon, malathion, parathion, and chlorpyrifos on the LiChrospher HPTLC plate (n = 4). After chromatographic separation, using a one-step HPTLC development process, and oxidation by N-bromosuccinimide, the plates were measured with an AChE inhibition assay using indoxyl acetate as substrate. AChE was sprayed onto the plates. The plates were scanned at 670 nm.

**Substances**	**Mean** R_F	**SD**	**rel.** R_F	**R**
Malaoxon	0.18	0.02		
Paraoxon	0.26	0.03	1.0	1.5
Chlorpyrifos-oxon	0.47	0.04	2.2	1.8
Malathion	0.59	0.05	1.1	1.3
Parathion	0.69	0.06	1.1	1.2
Chlorpyrifos	0.82	0.05	1.4	1.2

Tab. S6 Limits of detection (LOD) for malathion, oxidized malathion, malaoxon, chlorpyrifos, oxidized chlorpyrifos, chlorpyrifos-oxon, parathion, oxidized parathion, and paraoxon. An organothiophosphate (OTP) mix and an oxon mix were applied with different concentrations on LiChrosphere HPTLC plates. The application volume was 10 µL for the oxons and OTPs with following oxidation and 100 µL for the OTPs without following oxidation. After chromatographic separation by HPTLC, an AChE inhibition assay either with or without prior oxidation by N-bromosuccinimide was performed. The HPTLC plates were either immersed in AchE solution (left side) or AchE was sprayed onto the plates (right side). The peak heights were used for evaluation. The first detected peak at one of the applied concentrations with a signal-to-noise ratio ≥ 3 defines the LOD for the specific substance and respective method. The heights next to the respective peak defines the noise.

**Substances**	**LOD immersion method (ng/spot)**	**LOD spray method (ng/spot)**
Malathion	250	250
Oxidized malathion	0.25	0.1
Malaoxon	0.1	0.1
Chlorpyrifos	500	250
Oxidized chlorpyrifos	0.25	0.25
Chlorpyrifos-oxon	0.25	0.25
Parathion	500	100
Oxidized parathion	1.0	0.25
Paraoxon	0.5	0.5

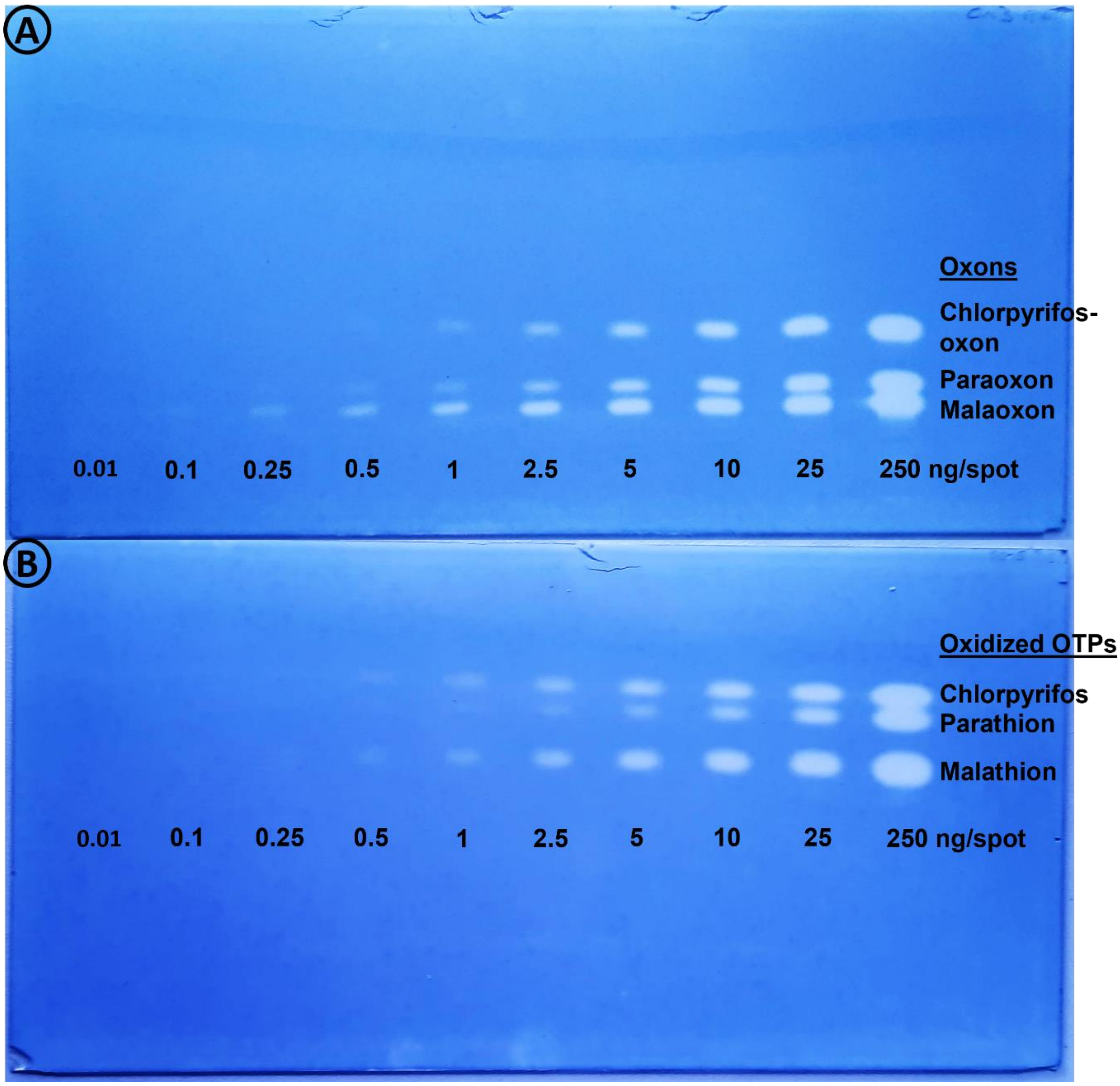

Fig. S4 Results of dose-response investigations for malathion, malaoxon, parathion, paraoxon, chlorpyrifos, and chlorpyrifos-oxon. (A) An oxon mix and (B) an organothiophosphate (OTP) mix were applied with amounts between 250 and 0.01 ng/spot on LiChrosphere HPTLC plates. The application volume was 10 µL. After chromatographic separation by HPTLC, an AChE inhibition assay with prior oxidation by N-bromosuccinimide was performed. AchE was sprayed onto the plates.

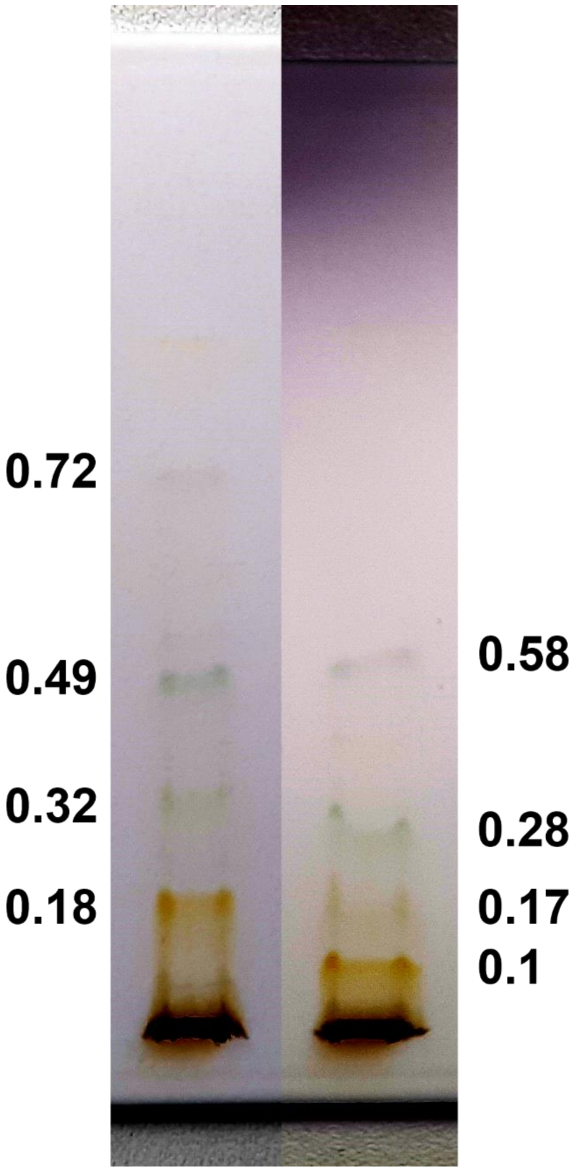

Fig. S5 Separation of an enriched combined sewer overflow sample with HPTLC on LiChrosphere plates. The application volume was 100 µL. Two automatic development procedures were used: A four-step development with increasing migration distance and elution power (right side), and a single-step development only using the last step of the four-step development (left side). Every step contains another amount of the used solvents. First step: 100% cyclohexane, second step: 90% cyclohexane and 10% dichloromethane, third step: 80% cyclohexane and 20% dichloromethane, and fourth step: 70% cyclohexane, 10% dichloromethane, and 20% acetone. Final migration distance was 80 mm. R_F values of separated bands are shown next to the images.
